# Supplementary material for: Causal role for sleep-dependent reactivation of learning-activated sensory ensembles for fear memory consolidation
Source: Nat Commun. 2021 Feb 22;12:1200. doi: 10.1038/s41467-021-21471-2 (PMC7900186; doi:10.1038/s41467-021-21471-2)
Supplement: Supplementary file 1 — Supplementary Information [file 41467_2021_21471_MOESM1_ESM.pdf]

<u>Extended Data Figure Legends:</u>

Extended Data Figure S1. ***Effects of post-conditioning sleep deprivation (SD) on pre-cue vs. cue-associated freezing behavior.*** Data show percent time spent in freezing behavior for freely-sleeping and SD mice at ZT12 testing, before (pre) and during presentation of shock (X°) and neutral (Y°) cues. Mice allowed *ad lib* sleep following conditioning (*n* = 19) froze significantly more during presentation of the shock cue (X°) than during presentation of the neutral cue (Y°), or during the pre-presentation interval for either cue (**** indicates $p < 0.0001$, Holm-Sidak *post hoc* test). SD mice (*n* = 16) froze significantly more during presentation of the shock cue (X°) than during the pre-presentation interval for either cue (** and * indicate $p = 0.002$ and $p = 0.01$, respectively, Holm-Sidak *post hoc* test) but did not discriminate between the two cues (X° vs. Y°, *N.S.,* Holm-Sidak *post hoc* test). Values indicate mean ± SEM.

Extended Data Figure S2. ***Both female and male mice show deficits in visually-cued fear memory following post-conditioning SD.*** **(a)** Male mice allowed *ad lib* sleep following conditioning froze significantly more to the shock cue (X°) than mice who were sleep deprived (* indicates $p = 0.019$, Holm-Sidak *post hoc* test; *n* = 12 and 9 mice for sleep and SD, respectively). Freely-sleeping male mice froze significantly more in response the shock cue than a neutral cue (*** indicates $p < 0.001$, Holm-Sidak *post hoc* test; *n* = 12 and 9 mice for sleep and SD, respectively). **(b)** Sleeping, but not SD, male mice showed discrimination for shock vs. neutral cues above chance (*** indicates $p = 0.001$, Wilcoxon signed rank test). **(c)** Female mice who were allowed *ad lib* sleep also showed higher freezing responses to the shock cue than the neutral cue (** indicates p = 0.007, Holm-Sidak *post hoc* test; *n* = 7 and 7 mice for sleep and SD, respectively). **(d)** Female mice allowed *ad lib* sleep showed discrimination in their responses to shock vs. neutral cues, while sleep deprived female mice did not (* indicates $p = 0.02$, Wilcoxon signed rank test vs chance; *n* = 7 and 7 mice for sleep and SD, respectively). Values indicate mean ± SEM.

Extended Data Figure S3. ***Effects of cue presentation order and shock and neutral cue orientation.*** **(a)** To test for effects of cue presentation order, mice were fear conditioned in Context A with presentation of an X° cue at ZT0. At ZT12, mice were tested in Context B and with either a Y° neutral cue followed by the X° shock cue, or with X° followed by Y°. **(b)** Mice showed greater discrimination between shock and neutral cues (with higher freezing to X°) when Y° was presented first. (** indicates $p = 0.01$, unpaired t–test between the two groups; *n* = 3 mice/group) Values indicate mean ± SEM. **(c)** To determine whether discrimination between shock and neutral cues was affected by selection of specific grating orientations, in a pilot study mice were conditioned to different shock cues (90° or 135°), and tested for responses to shock and a range of neutral cues (0°, 90°, or 135°). **(d-f)** Discrimination between shock and neutral cues was similar regardless of chosen orientations. Each graph shows freezing data for one mouse with different X° (shock) and Y° (neutral) cue orientations.

Extended Data Figure S4. ***Firing rate responses to optogenetic stimulation of TRAPed V1 neurons.*** **Top:** Traces of z-scored firing rate histograms for all significantly activated V1 neurons recorded from ChR2-expressing mice, showing timing of peak firing responses relative to blue

light pulses (starting at lag time 0). **Bottom:** Detail showing histograms for activated neurons with peak firing z-scores < 15.

Extended Data Figure S5. *State-specific targeting of optogenetic inhibition.* There were no significant differences in state coverage between different experimental groups (*N.S.*, two-way RM ANOVA for *n* = 8 no-opsin [no-inhibition] control mice, *n* = 8 mice cued to X° with subsequent inhibition, *n* = 7 mice cued to Y° with subsequent inhibition). In each group, light was delivered to V1 throughout most of REM sleep (93 ± 3%, 96 ± 1%, and 95 ± 3% of total REM, respectively) and NREM sleep (69 ± 3%, 75 ± 4%, and 79 ± 4% of total NREM, respectively) were covered. In each group there was a small amount of light delivery during wake, primarily during microarousals (19 ± 2%, 15 ± 2%, and 22 ± 3% of total wake, respectively). Values indicate mean ± SEM.

Extended Data Figure S6. *Sleep architecture and power during baseline and optogenetic inhibition.* **(a)** Representative traces of EEG classified as NREM sleep, REM sleep, and wake. **(b-d)** Percent of recording time spent in each state across recording periods and experimental groups (*n* = 8 mice/group). There were no significant differences in sleep time between groups (*N.S.*, two-way RM ANOVA). **(e-g)** Average bout length for each state across recording times and across experimental groups. There were no significant differences between groups (*N.S.*, two-way RM ANOVA). **(h-j)** Average power within NREM delta (0.5-4 Hz), NREM spindle (12-15 Hz), and REM theta (4-12 Hz) frequency bands across recording periods and experimental groups. There were no significant differences between groups (*N.S.*, two-way RM ANOVA). Values indicate mean ± SEM.

Extended Data Figure S7. *Effects of post-conditioning optogenetic inhibition on pre-cue vs. cue-associated freezing behavior.* Data show percent time spent in freezing behavior for *cfos::ArchT* mice at ZT12 testing, before (pre) and during presentation of shock and neutral cues. No-inhibition (non-opsin-expressing) controls (*n* = 8) and mice cued to Y° with subsequent optogenetic inhibition (*n* = 8) showed higher freezing responses to the shock cue vs. both pre-cue periods and the neutral cue (two-way RM ANOVA: main effect of optogenetic manipulation condition, $F = 14.333$, $p < 0.001$, main effect of orientation, $F = 9.2$, $p < 0.001$, optogenetic condition x orientation interaction, $F = 3.495$, $p = 0.005$; no-inhibition control - p = 0.01, 0.003, and < 0.001 for shock vs. pre-shock, neutral, and pre-neutral, respectively, Holm-Sidak *post hoc* test; Y°-cued inhibition – p = 0.002, < 0.00, and < 0.001 for shock vs. neutral, pre-shock, and pre-neutral, respectively, Holm-Sidak *post hoc* test). In contrast, mice cued to X° with subsequent optogenetic inhibition (*n* = 8) did not differ in freezing responses to the shock cue vs. either pre-cue periods or the neutral cue (*N.S.* for all comparisons, Holm-Sidak *post hoc* test). Mice cued to either X° or Y° with subsequent inhibition showed higher freezing responses to neutral cues and pre-cue periods relative to no-inhibition controls, indicative of generalization ($p < 0.001$ in X° cued mice vs. controls, and p = 0.004 and 0.04, for neutral and pre-cue periods respectively, in Y° cued mice vs. controls, Holm-Sidak *post hoc* test). *, **, and *** indicate $p = 0.01$, $p < 0.005$, and $p < 0.001$, respectively, vs. shock cue freezing. Values indicate mean ± SEM.

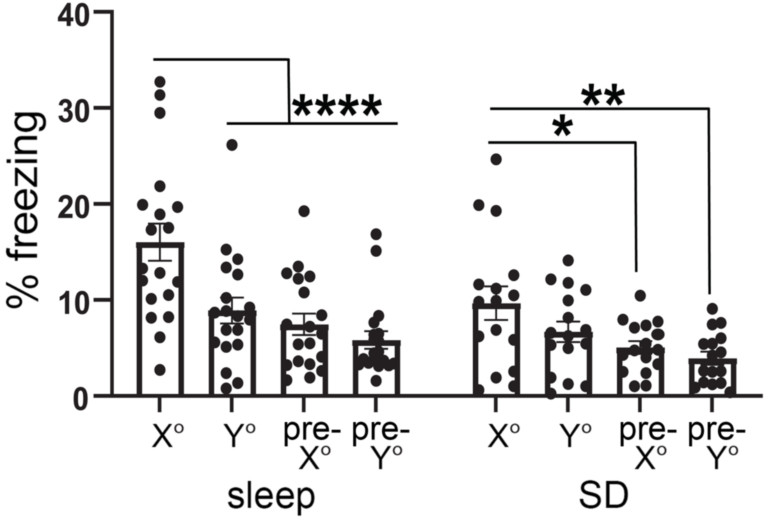

% freezing
40
30
20
10
0
****
**
*
X°
Y°
pre-X°
pre-Y°
sleep
X°
Y°
pre-X°
pre-Y°
SD

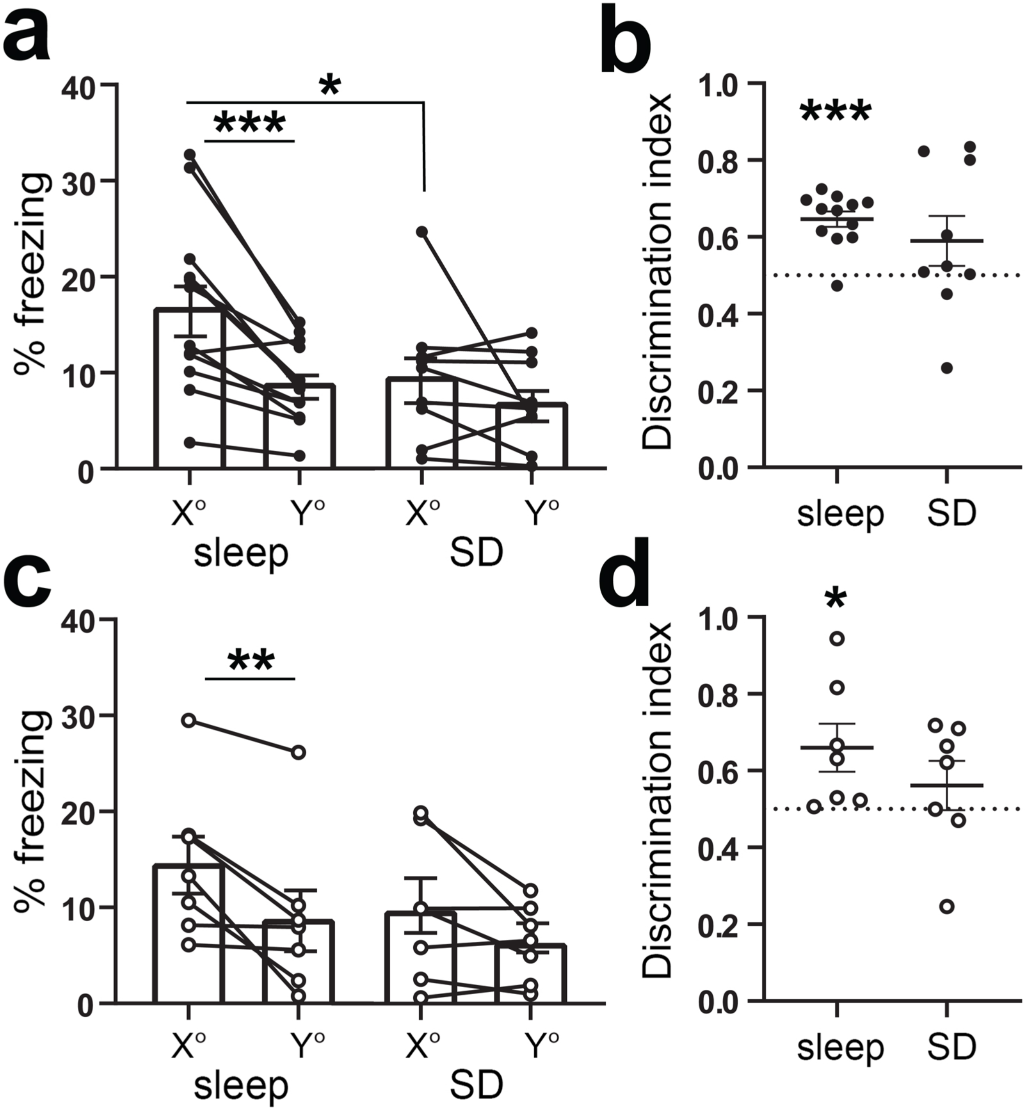
a
b
c
d
% freezing
Discrimination index
***
*
**
X°
Y°
sleep
SD
0
10
20
30
40
0.0
0.2
0.4
0.6
0.8
1.0

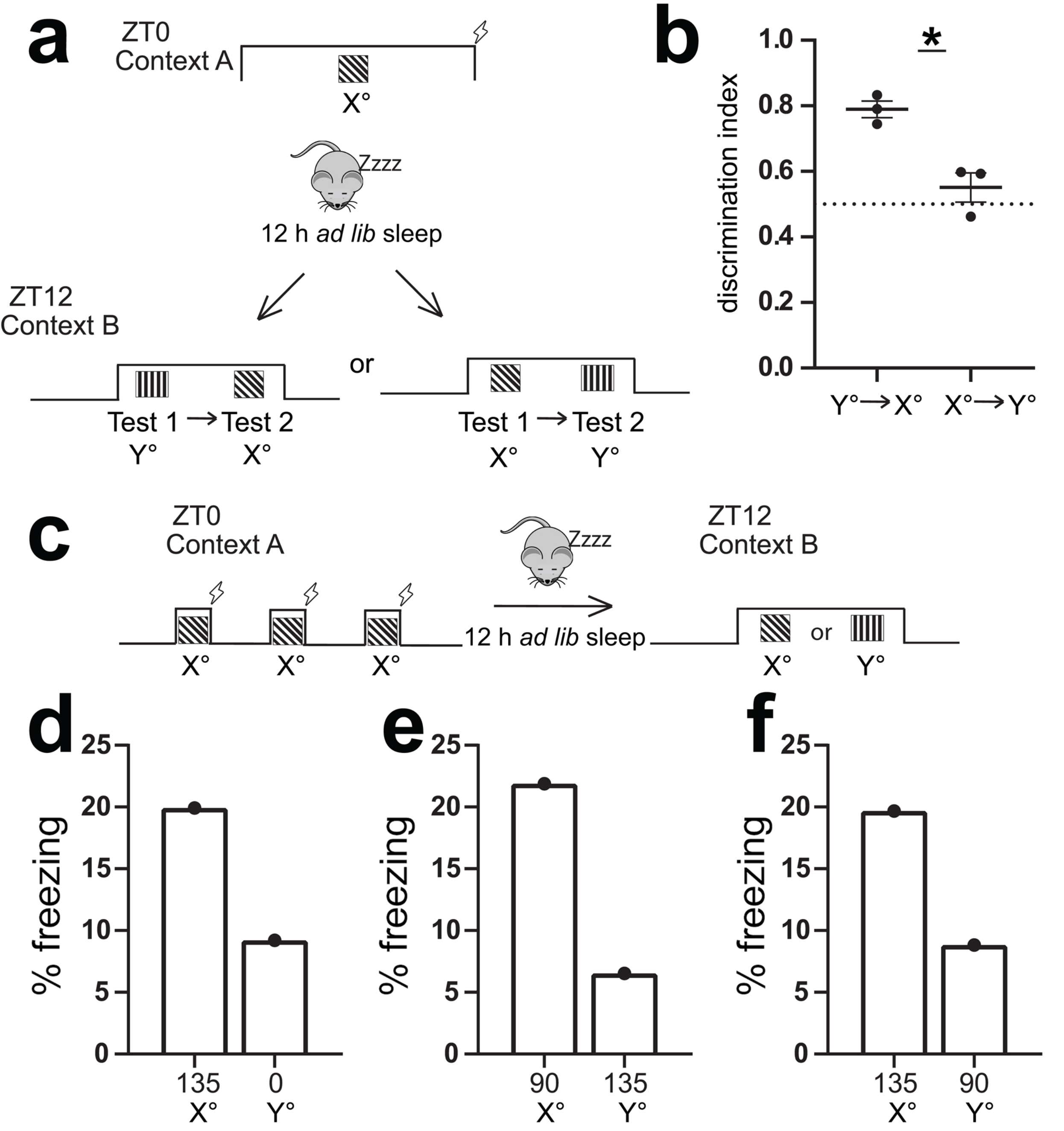
a
ZT0
Context A
X°
Zzzz
12 h ad lib sleep
ZT12
Context B
Test 1 → Test 2
Y° X°
or
Test 1 → Test 2
X° Y°
b
discrimination index
1.0
0.8
0.6
0.4
0.2
0.0
*
Y°→X°
X°→Y°
c
ZT0
Context A
X° X° X°
Zzzz
12 h ad lib sleep
ZT12
Context B
X° or Y°
d
% freezing
25
20
15
10
5
0
135 X°
0 Y°
e
% freezing
90 X°
135 Y°
f
% freezing
135 X°
90 Y°

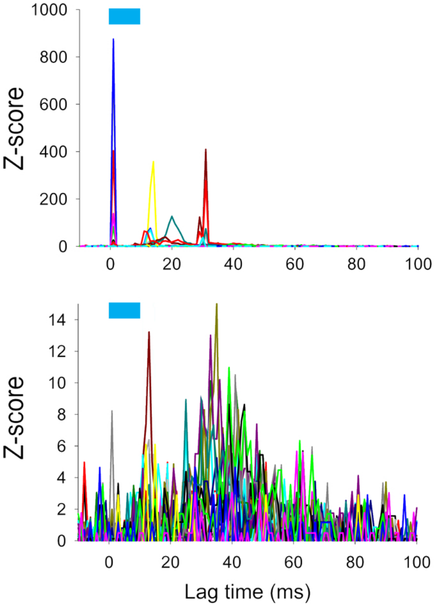

1000
800
600
400
200
0
Z-score
0
20
40
60
80
100
14
12
10
8
6
4
2
0
Z-score
0
20
40
60
80
100
Lag time (ms)

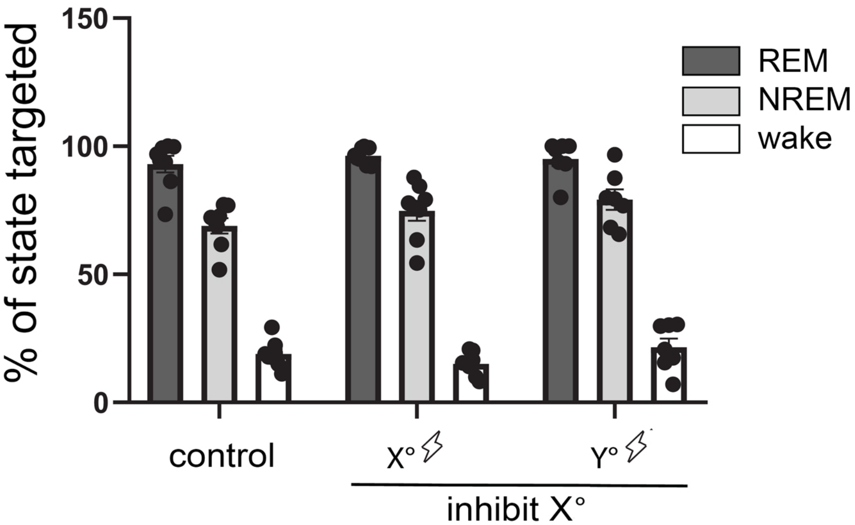

% of state targeted
150
100
50
0
REM
NREM
wake
control
X°
Y°
inhibit X°

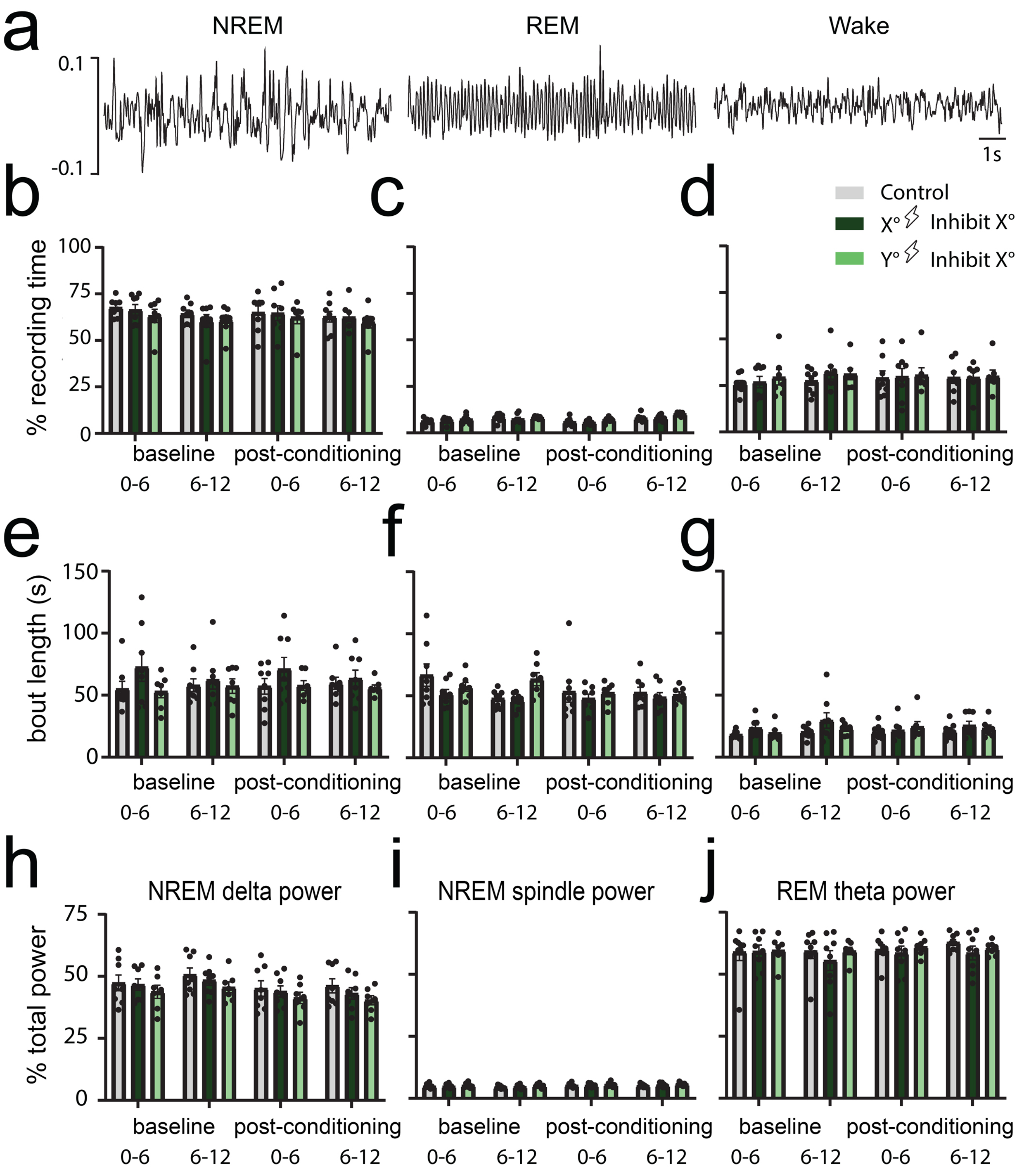

a
NREM
REM
Wake
0.1
-0.1
1s
b
c
d
Control
X° Inhibit X°
Y° Inhibit X°
% recording time
100
75
50
25
0
baseline
post-conditioning
0-6
6-12
0-6
6-12
e
f
g
bout length (s)
150
100
50
0
h
i
j
NREM delta power
NREM spindle power
REM theta power
% total power

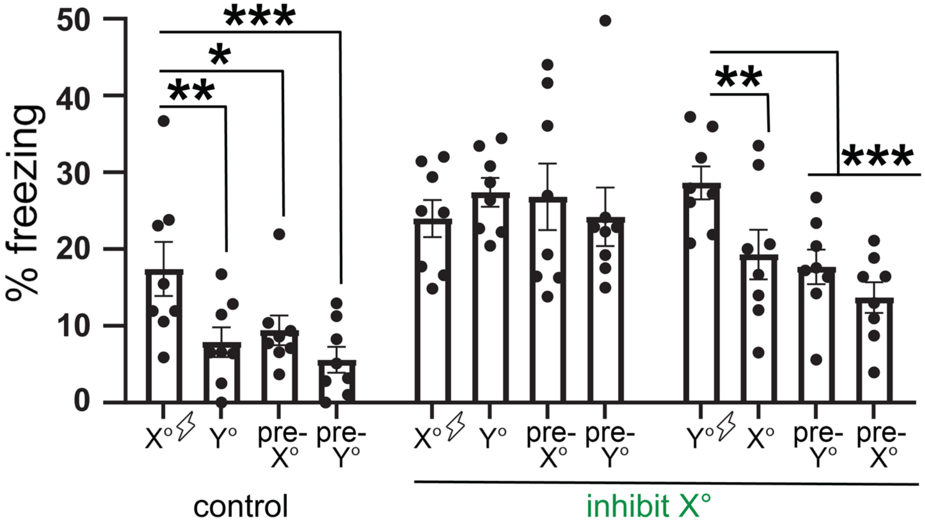

% freezing
50
40
30
20
10
0
***
*
**
**
***
X°
Y°
pre-X°
pre-Y°
X°
Y°
pre-X°
pre-Y°
Y°
X°
pre-Y°
pre-X°
control
inhibit X°
