# Supplementary material for: Causal role for sleep-dependent reactivation of learning-activated sensory ensembles for fear memory consolidation
Source: Nat Commun. 2021 Feb 22;12:1200. doi: 10.1038/s41467-021-21471-2 (PMC7900186; doi:10.1038/s41467-021-21471-2)
Supplement: Supplementary file 3 — Rerpoting Summary [file 41467_2021_21471_MOESM3_ESM.pdf]

nature research

Corresponding author(s): Brittany C. Clawson1, Emily J. Pickup1, Amy Ensing1, Laura Geneseo1, James Shaver1, John Gonzalez-Amoretti2, Meiling Zhao1, A. Kane York3, Femke Roig Kuhn1,4, Kevin Swift5, Jessy D. Martinez1, Lijing Wang1, Sha Jiang1, Sara J. Aton1#

Last updated by author(s): Dec 26, 2020

Reporting Summary

Nature Research wishes to improve the reproducibility of the work that we publish. This form provides structure for consistency and transparency in reporting. For further information on Nature Research policies, see our Editorial Policies and the Editorial Policy Checklist.

Statistics

For all statistical analyses, confirm that the following items are present in the figure legend, table legend, main text, or Methods section.

n/a	Confirmed	
☐	☒	The exact sample size (*n*) for each experimental group/condition, given as a discrete number and unit of measurement
☐	☒	A statement on whether measurements were taken from distinct samples or whether the same sample was measured repeatedly
☐	☒	The statistical test(s) used AND whether they are one- or two-sided<br>*Only common tests should be described solely by name; describe more complex techniques in the Methods section.*
☐	☒	A description of all covariates tested
☐	☒	A description of any assumptions or corrections, such as tests of normality and adjustment for multiple comparisons
☐	☒	A full description of the statistical parameters including central tendency (e.g. means) or other basic estimates (e.g. regression coefficient) AND variation (e.g. standard deviation) or associated estimates of uncertainty (e.g. confidence intervals)
☐	☒	For null hypothesis testing, the test statistic (e.g. *F*, *t*, *r*) with confidence intervals, effect sizes, degrees of freedom and *P* value noted<br>*Give P values as exact values whenever suitable.*
☐	☒	For Bayesian analysis, information on the choice of priors and Markov chain Monte Carlo settings
☐	☒	For hierarchical and complex designs, identification of the appropriate level for tests and full reporting of outcomes
☐	☒	Estimates of effect sizes (e.g. Cohen's *d*, Pearson's *r*), indicating how they were calculated

Our web collection on statistics for biologists contains articles on many of the points above.

Software and code

Policy information about availability of computer code

Data collection	Plexon Omniplex software was used to collect all electrophysiological data
Data analysis	Image J (FIJI - ImageJ 1.52P) was used for brain imaging analysis. Electrophysiological data were sorted using Offline Sorter (version 4, Plexon) and were analyzed using NeuroExplorer (version 4, Plexon) software. Statistical analysis was carried out using Prism (version 8) or Sigmaplot (version 14.0). Custom Matlab scripts were used for sleep scoring; this code is freely available from the Aton lab upon request.

For manuscripts utilizing custom algorithms or software that are central to the research but not yet described in published literature, software must be made available to editors and reviewers. We strongly encourage code deposition in a community repository (e.g. GitHub). See the Nature Research guidelines for submitting code & software for further information.

Data

Policy information about availability of data

All manuscripts must include a data availability statement. This statement should provide the following information, where applicable:

- Accession codes, unique identifiers, or web links for publicly available datasets
- A list of figures that have associated raw data
- A description of any restrictions on data availability

All data will be made available upon reasonable request.

Field-specific reporting

Please select the one below that is the best fit for your research. If you are not sure, read the appropriate sections before making your selection.

☒ Life sciences ☐ Behavioural & social sciences ☐ Ecological, evolutionary & environmental sciences

For a reference copy of the document with all sections, see nature.com/documents/nr-reporting-summary-flat.pdf

Life sciences study design

All studies must disclose on these points even when the disclosure is negative.

Sample size	Power analyses were used to determine appropriate sample sizes.
Data exclusions	Appropriate transgene expression was verified histologically for all mice where applicable. Inappropriate transgene expression (i.e., no transgene expression in experimental animals) was the only criterion used for data exclusion. This was applied to experiments where cfos-driven tdTomato, ChR2, or Arch expression was induced as part of the experimental design.
Replication	All attempts at replication were successful, with the exception of data exclusion due to lack of appropriate transgene expression.
Randomization	Mice were randomly assigned to experimental groups.
Blinding	Blinding of experimentalist during data collection was not possible, but all data analysis was done in a blinded manner. All behavioral scoring was carried out on coded video files by either two or three scorers blinded to treatment conditions. Sleep scoring and electrophysiological data analysis was done using coded files by an individual blinded to treatment, and validated by a second blinded scorer.

Reporting for specific materials, systems and methods

We require information from authors about some types of materials, experimental systems and methods used in many studies. Here, indicate whether each material, system or method listed is relevant to your study. If you are not sure if a list item applies to your research, read the appropriate section before selecting a response.

Materials & experimental systems

n/a	Involved in the study	
☐	☒	Antibodies
☒	☐	Eukaryotic cell lines
☒	☐	Palaeontology and archaeology
☐	☒	Animals and other organisms
☒	☐	Human research participants
☒	☐	Clinical data
☒	☐	Dual use research of concern

Methods

n/a	Involved in the study	
☒	☐	ChIP-seq
☒	☐	Flow cytometry
☒	☐	MRI-based neuroimaging

Antibodies

Antibodies used	Immunohistochemistry for cFos was carried out using rabbit-anti-cfos 1:1000 (Abcam; ab190289) and secondary donkey-anti-rabbit conjugated to Alexa Fluor 405 (1:200; Abcam; ab175651)
Validation	Appropriate detection of cFos was confirmed in a pilot study of cFos expression in V1 following a brief period of visual experience (as shown in Figure 2) vs. no experience (i.e., dark housing). As expected for detection of cFos, expression in V1 neurons was reduced after a period with no visual experience.

Animals and other organisms

Policy information about studies involving animals; ARRIVE guidelines recommended for reporting animal research

Laboratory animals	For all experiments, male and female mice were used between postnatal days 90 and 180. C57BL/6J mice (Jackson) were used for experiments outlined in Figure 1. For the remaining experiments, cfos-CREER mice (Guenthner et al 2013; B6.129(Cg)-Fostm1.1(cre/ERT2)Luo/J; Jackson) crossed to either B6.Cg-Gt(ROSA)26Sortm9(CAG-tdTomato)Hze/J, B6.Cg-Gt(ROSA)26Sortm32(CAG-COP4*H134R/EYFP)Hze/J, or B6.Cg-Gt(ROSA)26Sortm40.1(CAG-aop3/EGFP)Hze/J (Jackson) mice to induce CRE recombinase-mediated expression of tdTomato, ChR2, or ArchT. Housing was maintained at 18-23°C and 40-60% relative humidity throughout all experiments, and a 12 h: 12 h light:dark cycle was maintained, with the exception of dark housing following tamoxifen or corn oil administration.

Wild animals	No wild animals were used in the study.
Field-collected samples	No field collected samples were used in the study.
Ethics oversight	All procedures described in the manuscript were reviewed and approved by the University of Michigan Institutional Animal Care & Use Committee.

Note that full information on the approval of the study protocol must also be provided in the manuscript.
